# Routine free thyroxine reference intervals are suboptimal for monitoring children on thyroxine replacement therapy and target intervals need to be assay-specific

**DOI:** 10.1038/s41598-019-55690-x

**Published:** 2019-12-13

**Authors:** Elizabeth Wheeler, Kay Weng Choy, Lit Kim Chin, Nilika Wijeratne, Alan McNeil, Tina Yen, Susan Matthews, David Deam, Zhong Lu, Tze Ping Loh, James Doery, Philip Bergman

**Affiliations:** 1 Department of Paediatric and Adolescent Endocrinology and Diabetes, Monash Children’s Hospital, Clayton, Australia; 2 Monash Pathology, Monash Medical Centre, Clayton, Australia; 3 Dorevitch Pathology, Heidelberg, Australia; 4 Department of Medicine, Monash University, Clayton, Australia; 5 The Royal Children’s Hospital Laboratory Services, Parkville, Australia; 6 Australian Clinical Labs, Clayton, Australia; 7 Melbourne Pathology, Parkville, Australia; 8 Department of Laboratory Medicine, National University Hospital, Singapore, Singapore; 9 Department of Paediatrics, Monash University, Clayton, Australia

**Keywords:** Diagnostic markers, Thyroid diseases

## Abstract

Central hypothyroidism is a condition where there is (qualitatively or quantitatively) TSH deficiency, leading to reduced thyroid hormone production. In such patients, serum TSH does not accurately reflect the adequacy of thyroxine replacement, as the log-linear relationship between thyrotropin (TSH) and free thyroxine (FT4) is lost. We aimed to prospectively determine the optimal physiological FT4 treatment range for children treated for primary hypothyroidism, based on their serum TSH concentrations. This information could be used to guide optimal therapy for all children on thyroxine replacement, including those with central hypothyroidism. In total, sixty children (median age: 11 years, range: 11 months to 18 years) were recruited over 21 months. They were prescribed a stable dose of thyroxine for at least 6–8 weeks prior to a thyroid function test that consisted of serum TSH, FT4 and free triiodothyronine (FT3) measurements. The serum sample for the thyroid function tests was collected before ingestion of the daily dose, i.e. the trough concentration, and measured using Beckman Coulter UniCel DxI 800 instrument, Siemens Advia Centaur, Roche Cobas, Abbott Architect, Ortho Clinical Diagnostics Vitros 5600 (Ortho-Clinical Diagnostics, Raritan, NJ) platforms. The FT4 and FT3 reference intervals showed significant inter-method difference. The lower limit of the FT4 reference intervals were generally shifted mildly higher when the TSH concentration of the children were restricted from 0.5–5.0 mIU/L to 0.5–2.5 mIU/L. By contrast, the upper limit of the FT3 and FT4 reference intervals were relatively stable for the different TSH concentrations. Assay-specific target ranges for optimal thyroxine therapy are required until FT4 assay standardisation is realised.

## Introduction

Thyroid stimulating hormone (TSH) is produced by the anterior pituitary gland and drives the production of thyroid hormone in the thyroid gland. It has a log-linear relationship with free thyroxine (FT4) concentration in blood. A small change in FT4 concentration will lead to an exponential change in TSH concentration. As such, TSH is considered the most sensitive biomarker for thyroid dysfunction. It is also the biomarker of choice for monitoring thyroid hormone replacement, where inadequate replacement is reflected by an increased serum TSH^1–3^.

Central hypothyroidism is a condition where there is (qualitatively or quantitatively) TSH deficiency, leading to reduced thyroid hormone production. Achieving adequate thyroxine replacement in patients with central hypothyroidism remains a challenge^1^ as the log-linear relationship between TSH and FT4 is lost in such patients. Consequently, serum TSH does not accurately reflect the adequacy of thyroxine replacement and has a limited role in such setting.

Given the limitations with TSH, serum FT4 measurement is the alternative biochemical monitor of thyroxine therapy adequacy in patients with central hypothyroidism^4^. However, the optimal target FT4 concentration for this group of patients is debated with some authors suggesting target FT4 concentration in the middle of the reference intervals^5^, while others advocating the upper half^6,7^. Yet, others consider the optimal thyroxine doses for central hypothyroidism to be similar to those for primary hypothyroidism^8^. The European Society for Paediatric Endocrinology Consensus Guidelines on primary (congenital) hypothyroidism recommend maintaining TSH in the age-specific reference range and serum FT4 concentrations in the upper half of the age-specific reference range^4^.

We aimed to prospectively determine the optimal physiological FT4 treatment range for children treated for primary hypothyroidism, based on their serum TSH concentrations. This information could be used to guide optimal therapy for all children on thyroxine replacement, including those with central hypothyroidism. Given the lack of standardisation between different thyroid assays, we also investigated if the target range would vary across different thyroid hormone assays.

## Patients and Methods

Children (defined as ≤18 years of age) with primary hypothyroidism, who were attending Monash Children’s Hospital, were prospectively recruited into this study following provision of informed consent from the parents. They were prescribed a stable dose of thyroxine for at least 6–8 weeks prior to a thyroid function test that consisted of serum TSH, FT4 and free triiodothyronine (FT3) measurements. This study design was approved by the Monash Health Human Research Ethics Committee (reference number: 15509 L). All methods were performed in accordance with the relevant guidelines and regulations.

At Monash Children’s Hospital, the thyroid function tests were routinely measured on the Beckman Coulter UniCel DxI 800 instrument (Beckman Coulter, Miami, FL). The serum sample for the thyroid function tests was collected before ingestion of the daily dose, i.e. the trough concentration, therefore at least 14 hours post last ingestion. The thyroid function tests were performed within four hours of sample collection. Leftover serum samples were stored at −70 °C. The archived samples were then subjected to additional TSH, FT4 and FT3 measurements within 12 months using laboratory instruments from four other major manufacturers: Siemens Advia Centaur (Siemens Healthcare, Erlangen, Germany), Roche Cobas (Roche Diagnostics, Indianapolis, IN), Abbott Architect (Abbott Laboratories, Abbott Park, IL), Ortho Clinical Diagnostics Vitros 5600 (Ortho Clinical Diagnostics, Raritan, NJ) at three collaborating laboratories in Victoria, Australia.

### Statistical analysis

The FT3 and FT4 results of the children were categorised into two groups based on their TSH measurement: 0.5–2.5 mIU/L (the lower half of the reference intervals in use at Monash Children’s Hospital) and 0.5–5.0 mIU/L (the entire reference intervals), and analysed separately. FT3 and FT4 results falling outside of the above TSH intervals were excluded from further analysis. The remaining FT3 and FT4 results were subjected to outlier detection by Reed’s test, followed by assessment of normality by Shapiro-Wilk test. Thyroid hormone results that failed the Shapiro-Wilk test were transformed to approximate normal distribution. The reference intervals were derived using the conventional parametric approach and the robust approach with bootstrapping (with 100,000 iterations and fixed seed), according to the CLSI C28-A3 recommendations. Similar analysis was performed for results obtained by the other laboratory instruments. The statistical analyses were performed using Microsoft Excel and MedCalc.

## Results

**Figure 1:**
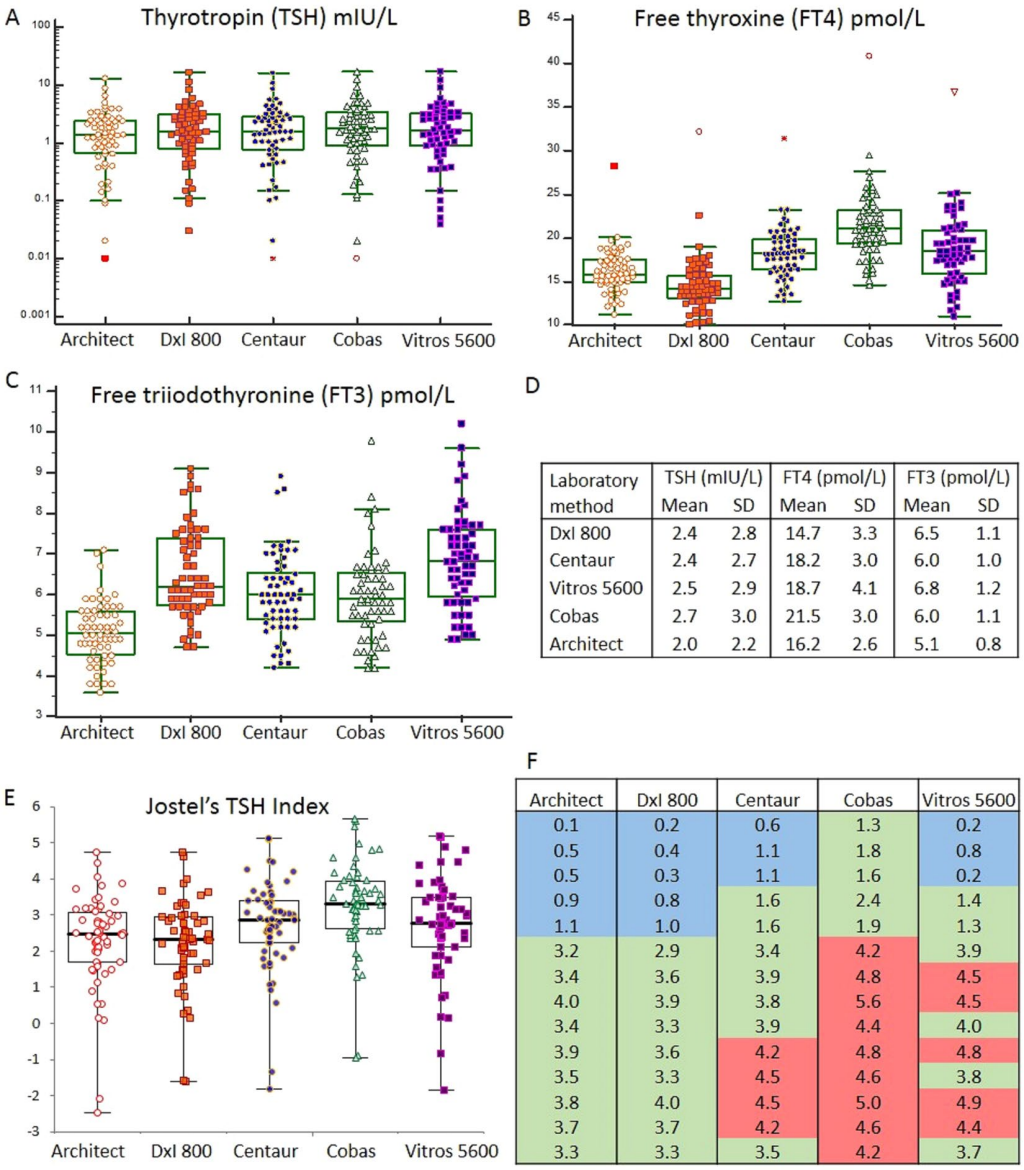
Distributions of the TSH (panel A), FT4 (panel B) and FT3 (panel C) and the average results of the sixty children (panel D). TSH results were presented in logarithmic scale. Distribution of Jostel’s TSH index (calculated by [ln(TSH) + 0.1345 = FT4], panel E), and 14 children with discordant interpretation based on a reference interval of 1.3–4.1 (panel F, cells shaded in blue were <1.3, in green were 1.3–4.1, in red were >4.1).

In total, sixty children (16 boys, 27%; 44 girls, 73%) were recruited over 21 months. The demographic and clinical details of the children included in this study are summarised in Table 1. The distributions of the TSH, FT3 and FT4 results of the sixty children are shown in Fig. 1. The overall mean of the TSH were broadly similar among the different manufacturer, except the Abbott Architect method, which produced the lowest averaged result. On the other hand, FT4 was highest when measured using the Roche Cobas method and lowest using the Abbott Architect method. The Abbott Architect also produced the lowest average FT3.

**Table 1:** Basic demographic and clinical details of children included in this study.

	Median	Q1-Q3	Min	Max
Age	11.0	6–13	0.9	18.0
Height (m)	1.42	1.12–1.60	0.71	1.76
Body weight (kg)	33	19–54	9	88
BMI (kg/m^2^)	18.0	16.3–21.5	13.3	35.6
1st Dose (μg)	100	50–100	25	200
2nd Dose (μg)	50	25–125	0	200
Total L-T4 per week (μg)	600	375–800	175	1,400
LT-4 dose (μg) per kg per week	17	14–21	6	60
**Causes of hypothyroidism**	**Frequency**	**Percentage (%)**		
Congenital hypothyroidism	32	53		
Lingual thyroid	6	10		
Hypothyroidism with negative autoantibodies	5	8		
Autoimmune thyroiditis	14	23		
Post thyroidectomy/radiotherapy	3	5

The reference intervals for FT3 and FT4 derived from children with TSH concentrations of 0.5–5.0 mIU/L and 0.5–2.5 mIU/L are summarised in Tables 2 and 3, respectively. The FT4 and FT3 reference intervals showed significant inter-method difference. The lower limit of the FT4 reference intervals were generally shifted mildly higher when the TSH concentration of the children were restricted from 0.5–5.0 mIU/L to 0.5–2.5 mIU/L. By contrast, the upper limit of the FT3 and FT4 reference intervals were relatively stable for the different TSH concentrations.

**Table 2:** FT4 and FT3 reference intervals derived using different laboratory instruments in children with TSH concentration 0.5–2.5 mIU/L.

Statistical Parameters	FT4, pmol/L					FT3, pmol/L				
	DxI 800	Centaur	Vitros 5600	Cobas	Architect	DxI 800	Centaur	Vitros 5600	Cobas	Architect
TSH < 0.5 mIU/L, n excluded	11	11	12	11	12	11	11	12	11	12
TSH > 2.5 mIU/L, n excluded	21	19	24	22	14	21	19	24	22	14
Outlier by Reeds test	0	0	0	0	0	0	0	0	0	0
Final n	28	30	24	27	34	28	30	24	27	34
Normality by Shapiro-Wilk test	Accept	Accept	Accept	Accept	Accept	Reject	Accept	Accept	Accept	Accept
Transformation	None	None	None	None	None	Logarithmic	None	None	None	None
Minimum	10.4	12.8	11.7	15.2	11.2	4.9	4.2	4.9	4.2	3.8
Maximum	17.5	23.2	23.1	25.4	19.2	8.7	7.3	8.9	7.0	7.0
Mean	14.0	17.9	18.0	20.8	15.8	6.4	5.7	6.7	5.7	5.1
Median	14.1	18.2	18.4	21.0	15.8	6.1	5.9	6.8	5.9	5.1
SD	1.7	2.2	2.7	2.6	1.8	NA		1.0	0.7	0.7
**Reference intervals (normal)**										
Lower limit (2.5th percentile)	10.6	13.6	12.6	15.8	12.2	4.7	4.3	4.7	4.4	3.7
90% CI	9.6 to 11.5	12.5 to 14.8	11.0 to 14.2	14.4 to 17.2	11.3 to 13.1	4.3 to 5.1	3.9 to 4.7	4.1 to 5.3	4.0 to 4.8	3.4 to 4.1
Upper limit (97.5th percentile)	17.4	22.2	23.4	25.8	19.3	8.7	7.2	8.8	7.1	6.4
90% CI	16.4 to 18.3	21.0 to 23.4	21.8 to 25.0	24.4 to 27.2	18.4 to 20.2	8.0 to 9.5	6.8 to 7.6	8.1 to 9.4	6.7 to 7.5	6.1 to 6.7
**Robust method (CLSI C28-A3)**										
Lower limit	10.5	13.4	12.7	15.7	12.2	4.4	4.2	4.5	4.4	3.6
90% CI	9.4 to 11.5	12.1 to 14.7	10.5 to 14.6	14.1 to 17.3	11.2 to 13.2	4.1 to 4.8	3.8 to 4.7	3.9 to 5.2	3.9 to 4.9	3.3 to 4.0
Upper limit	17.8	22.5	24.4	26.4	19.8	8.7	7.4	8.9	7.3	6.5
90% CI	16.7 to 18.6	21.2 to 23.7	22.6 to 25.9	24.9 to 27.7	18.8 to 20.6	7.8 to 9.8	7.01 to 7.8	8.3 to 9.5	6.9 to 7.7	6.1 to 6.8

**Table 3:** FT4 and FT3 reference intervals derived using different laboratory instruments in children with TSH concentration 0.5–5.0 mIU/L.

Statistical Parameters	FT4, pmol/L					FT3, pmol/L				
	DxI 800	Centaur	Vitros 5600	Cobas	Architect	DxI 800	Centaur	Vitros 5600	Cobas	Architect
TSH < 0.5 mIU/L, n excluded	11	11	12	11	12	11	11	12	11	12
TSH > 5.0 mIU/L, n excluded	5	5	4	6	3	5	5	4	6	3
Outlier by Reeds test	0	0	0	0	0	0	0	0	0	0
Final n	44	44	44	43	45	44	44	44	43	45
Normality by Shapiro-Wilk test	Accept	Accept	Accept	Accept	Accept	Reject	Accept	Accept	Accept	Accept
Transformation	None	None	None	None	None	Logarithmic	None	None	None	None
Minimum	10.1	12.8	11.0	14.6	11.2	4.7	4.2	4.9	4.2	3.6
Maximum	19.0	23.2	25.0	27.0	20.1	9.1	8.5	9.2	8.4	7.0
Mean	13.9	17.6	17.7	20.5	15.7	6.4	5.8	6.7	5.9	5.0
Median	14.0	18.0	18.0	20.6	15.6	6.1	5.9	6.8	5.9	5.0
SD	2.0	2.3	3.1	2.8	1.9	NA	0.9	1.1	1.0	0.7
**Reference intervals (normal)**										
Lower limit (2.5th percentile)	10.0	13.1	11.5	15.1	11.8	4.6	4.1	4.6	4.0	3.6
90% CI	9.1 to 10.8	12.1 to 14.1	10.2 to 12.9	13.8 to 16.3	11.0 to 12.7	4.3 to 5.0	3.7 to 4.5	4.1 to 5.0	3.6 to 4.4	3.4 to 3.9
Upper limit (97.5th percentile)	17.8	22.1	23.8	25.9	19.5	8.8	7.6	8.8	7.7	6.4
90% CI	17.0 to 18.7	21.1 to 23.0	22.4 to 25.1	24.7 to 27.1	18.6 to 20.3	8.2 to 9.5	7.2 to 8.0	8.3 to 9.3	7.3 to 8.1	6.1 to 6.7
**Robust method (CLSI C28-A3)**										
Lower limit	9.8	12.8	11.3	14.9	11.7	4.4	4.0	4.4	3.8	3.5
90% CI	8.9 to 10.8	11.9 to 14.0	9.9 to 13.0	13.7 to 16.2	10.9 to 12.6	4.1 to 4.8	3.6 to 4.5	4.0 to 5.0	3.4 to 4.3	3.2 to 3.9
Upper limit	17.9	22.3	24.1	26.2	19.7	8.9	7.6	8.9	7.7	6.5
90% CI	17.1 to 18.8	21.3 to 23.2	22.9 to 25.4	25.0 to 27.3	18.8 to 20.5	8.1 to 9.6	7.2 to 8.0	8.4 to 9.3	7.3 to 8.2	6.2 to 6.8

The FT4 reference intervals derived from children with TSH of 0.5–5.0 mIU/L using the robust method in this study were compared to previously published data, which are summarised in Table 4.

**Table 4:** Summary of existing paediatric reference intervals for FT4 using direct sampling approach.

	Published paediatric reference intervals			This study (11 months − 18 years)
	Source	Age	Reference intervals	
Beckman	Adeli *et al*.^14^	20 days–<3 years	9.5–17.8	9.8–17.9
		3–19 years	7.8–13.6	
Siemens	Kapelari *et al*.^23^	1–12 months	9.2–25.3	12.8–22.3
		1–5 years	10.5–22.4	
		6–10 years	10.6–20.9	
		11–14 years	10.4–21.4	
		15–18 years	10.6–22.6	
Roche	Taylor *et al*.^24^	7 years	12.7–19.3	11.3–24.1
		15 years	11.9–20.3	
Abbott	Adeli *et al*.^14^	30 days–<1 year	11.4–21.9	14.9–26.2
		1–<19 years	11.4–17.6	
Vitros	Adeli *et al*.^14^	2 weeks–<5 years	11.4–29.2	11.7–19.7
		5–<19 years	9.7–17.1	

## Discussion

Thyroid hormones play a key role in the growth and development of children. Inadequate thyroid hormone in children is associated with mental and growth retardation^4^. Hence, it is important to ensure adequate replacement of these hormones in children who are deficient. For this purpose, guidelines have generally recommended to aim for a TSH concentration in the lower half of the reference interval, or (typically) <2.5 mIU/L, for primary hypothyroidism^9,10^. In central hypothyroidism, TSH is not able to be used for analysis, therefore measurement of thyroxine only can be relied upon.

Using TSH of 0.5–2.5 mIU/L as an inclusion criterion, we derived FT3 and FT4 reference intervals for a cohort of children who were receiving thyroxine replacement for primary hypothyroidism. At the same time, we also examined the impact of widening the TSH inclusion criteria to 0.5–5.0 mIU/L, which produced correspondingly wider FT3 and FT4 reference intervals. Nevertheless, the wider thyroid hormone reference limits did not fall outside of the 90% confidence intervals of those obtained from children with TSH 0.5–2.5 mIU/L. This suggested no significant difference in reference limits between these two groups of children. This observation corroborates with studies demonstrating no difference in health outcomes in patients whose target TSH concentration was in the lower half (0.4–2.0 mIU/L) compared to those with higher target TSH concentration (2.0–4.0 mIU/L)^11^. One reason the lower half of TSH reference interval is used in paediatric patients is to allow for dose adjustment anticipating increasing requirements for growth.

This study showed that thyroid hormone assays remain poorly standardised at present. When controlled for TSH, the lower and upper reference limits for FT4 differed by close to 50% between the assays with the lowest (Beckman Coulter DxI 800) and highest (Ortho Clinical Diagnostics Vitros 5600) measurements. In view of the poor standardisation, it is necessary for laboratories to apply method-specific reference intervals that best suit the population they serve. There is an ongoing effort within the International Federation for Clinical Chemistry and Laboratory Medicine (IFCC) to standardise thyroid hormone assays^12^. It is hoped that when standardisation is realised, a common reference interval may be adopted across assays^13^.

The reference intervals for four of the five FT4 assays (Beckman Coulter, Roche Cobas, Abbott Architect and Ortho Vitros) were generally higher than those reported for healthy children. For example, for the Beckman Coulter assay, in our subjects with TSH concentrations in the range of 0.50–2.50 mIU/L the upper limit of the FT4 interval was approximately 28% higher (FT4, 17.4 pmol/L) than the published upper reference limit for FT4 of 13.6 pmol/L^14^. Similarly, for the Ortho Vitros assay, the FT4 upper reference limit of 23.8 pmol/L derived from our subjects was approximately 40% higher than the published upper reference limit of 17.1 pmol/L (age 5–<19 years)^14^. Unlike the TSH assays (Fig. 1), this study demonstrated that FT4 measurements clearly varied between the five different major assays when the same samples are measured. The lack of standardisation also affected interpretation of calculated parameters of thyroid homeostasis, such as Jostel’s TSH index in 14 of the 60 (23%) children (Fig. 1).

Our study suggests that the FT4 target ranges for children on thyroxine replacement is likely higher than the reference intervals derived from healthy children. This finding, which requires confirmation from larger studies, supports the recommendation of titrating thyroxine replacement to the mid or upper range of the ‘routine’ reference intervals^4^, since that is likely the FT4 concentration at which TSH will be kept at 0.5–2.5 mIU/L. When recommendations are made on target TSH interval (e.g. lower half of the reference interval for primary hypothyroidism) or target FT4 interval (e.g. upper or middle of the reference interval for central hypothyroidism), it raises important issues regarding the source and quality of the reference intervals.

In establishing reference intervals, some considerations include the extent required to exclude all thyroid disorders, age-partitioning in the paediatric population and biological variation (any significant variation with time of day)^15,16^. Recent studies have also proposed ethnic-specific reference intervals^17,18^. It is not surprising that reference intervals for thyroid hormones vary between assays and even within the same assays^19–21^. The relationship between TSH and FT4 is complex and nonlinear^22^. Direct derivation of target thyroid hormone ranges from patients treated with steady dose of thyroxine may mitigate some of these considerations.

There are some limitations to this study. The number of subjects included in this study is relatively small. Care must be exercised when interpreting its results, which should be considered as preliminary until evidence from larger studies are available. However, recommended robust methods including bootstrapping were applied to improve the quality of the statistical analysis. The relatively small number of subjects also precluded more detailed analysis with age partitioning. Future studies should include larger number of subjects to examine the impact of age-related changes. This is especially important since thyroid hormone concentration and deiodinase activity are age-dependent. Additionally, each child was only sampled once for this study. A higher number of sampling for each child will improve the estimation of her point of homeostasis. Finally, this study only examined the biochemical surrogates of the children. Future studies may recruit a larger number of children and follow them up prospectively for other clinical outcomes including growth and intellectual development.

In conclusion, children who are on thyroxine replacement for primary hypothyroidism require a target serum FT4 concentration range higher than the routine laboratory reference interval derived for the general population. We propose that this target range can also be applied on patients with central hypothyroidism. The work of TSH assay harmonisation and FT4 standardisation is being actively pursued by IFCC^12^. In the meantime, assay-specific target ranges for optimal thyroxine therapy are required until FT4 assay standardisation is realised.

## Data Availability

The data is available upon request from the authors.
